# Metformin attenuates high glucose-induced injury in islet microvascular endothelial cells

**DOI:** 10.1080/21655979.2022.2033411

**Published:** 2022-02-09

**Authors:** Wenyu Zou, Bingkun Liu, Yulu Wang, Fangbin Shi, Shuguang Pang

**Affiliations:** a Department of endocrinologyEndocrinology, Jinan Central Hospital, Cheeloo College of Medicine, Shandong University, Jinan, China; b Department of Cardiology, Yidu Central Hospital of Weifang, Weifang, China; c Department of Internal Medicine, Weifang Medical University, Weifang, China

**Keywords:** Metformin, proliferation, islet endothelium, apoptosis, oxidative stress

## Abstract

As one of the most frequently prescribed antidiabetic drugs, metformin can lower glucose levels, improve insulin resistance manage body weight. However, the effect of metformin on islet microcirculation remains unclear. In the present study, to explore the effect of metformin on islet endothelial cells and investigated the underlying mechanism, we assessed the effects of metformin on islet endothelial cell survival, proliferation, oxidative stress and apoptosis. Our results suggest that metformin stimulates the proliferation of pancreatic islet endothelial cells and inhibits the apoptosis and oxidative stress caused by high glucose levels. By activating farnesoid X receptor (FXR), metformin increases the expression of vascular endothelial growth factor-A (VEGF-A) and endothelial nitric oxide synthase (eNOS), improves the production of nitric oxide (NO) and decreases the production of ROS. After the inhibition of FXR or VEGF-A, all of the effects disappeared. Thus, metformin appears to regulate islet microvascular endothelial cell (IMEC) proliferation, apoptosis and oxidative stress by activating the FXR/VEGF-A/eNOS pathway. These findings provide a new mechanism underlying the islet-protective effect of metformin.

## GRAPHICAL ABSTRACT

**Figure:**
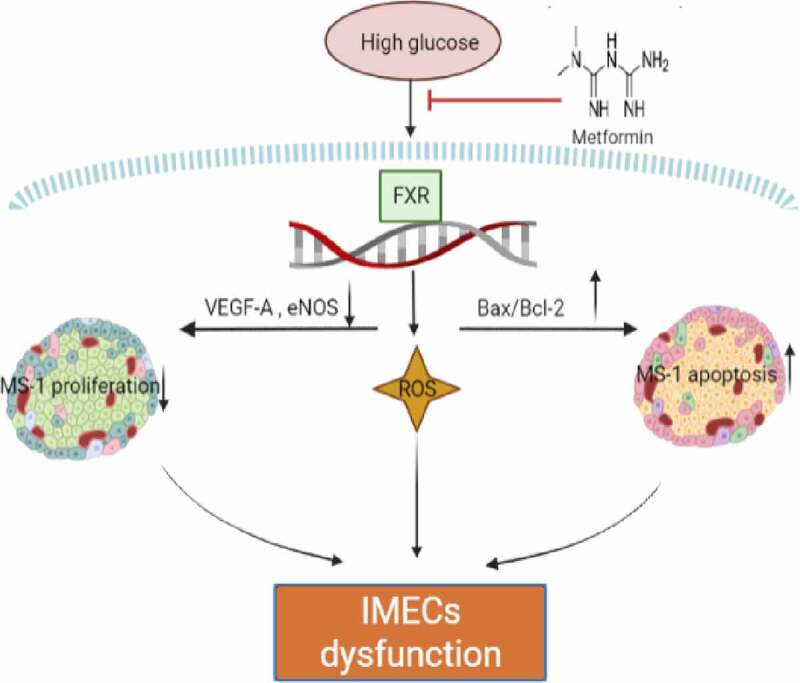

## 1. Introduction

Type 2 diabetes mellitus (T2DM) is a chronic disease characterized by high blood glucose levels and is often associated with insufficient insulin production and/or insulin resistance. Currently, diabetes is one of the major threats to human health worldwide. It is estimated that the number of patients with diabetes worldwide will exceed 693 million by 2045 [1]. Although the pathogenesis of diabetes used to be considered to involve inadequate insulin production due to the destruction of β cells, recent findings have suggested that the disruption of pancreatic islet microcirculation can contribute to the pathogenesis and development of diabetes [2].

In fact, disruption of the islet microcirculation may lead to inadequate nutrient supply due to fluctuations in glucose levels, resulting in islet dysfunction [3]. Previous studies have shown that this islet endothelial dysfunction may lead to impaired insulin release from β cells. In patients with T2DM, islet capillaries in the pancreas become thick and fragmented [4]. Changes in the morphology of islet capillaries have also been described in a variety of diabetic rodent models [5], and these changes are accompanied by a significantly reduced number of endothelial cells, endothelial cell thickening, and the loss of endothelial openings.

Metformin has been used as a first-line hypoglycemic drug for more than 60 years. Increased attention is being paid to metformin due to its ability to reduce insulin resistance, affecting glucose and lipid metabolism, and due to its anti-inflammatory and antitumor properties [6]. In addition, metformin exerts a cardioprotective effect independent of its hypoglycemic effect. However, no studies have examined the effect of metformin on islet microvascular endothelial cell (IMEC).

Farnesoid X receptor (FXR) belongs to the nuclear receptor superfamily and contributes to a variety of physiological metabolic processes, such as lipid, fatty acid and glucose metabolism [7]. In fact, it is also closely related to angiogenesis. Previous studies have shown that decreased FXR expression obstructs angiogenesis [8]. Vascular endothelial growth factor A (VEGF-A) is a major regulator of islet vascularization and revascularization [9,10]. The loss of VEGF-A function leads to insufficient angiogenesis and decreased glucose tolerance in islets, and the restoration of VEGF-A levels in islets after via its overexpression can induce β cell regeneration [11,12]. Nitric oxide (NO), generated by Endothelial nitric oxide synthase (eNOS), a downstream protein of VEGF-A, functions to regulate microcirculation and vascular permeability [11,12], and relaxes perivascular smooth However, it is unclear whether metformin affects the FXR-VEGF-A-eNOS signaling pathway in cultured MS-1 (a cell line derived from mouse IMECs) cells.

This study aims to investigate the effects of metformin on proliferation and apoptosis and to explore the molecular mechanisms underlying the effects of metformin in ameliorating islet microvascular endothelial cell dysfunction in vitro. In the present study, we hypothesize that metformin could promote MS-1 cell proliferation and inhibit the oxidative stress and apoptosis caused by high glucose (HG) levels by modulating FXR.

## 2. Materials and methods

### 2.1. Reagents

Metformin, glucose and fetal bovine serum (FBS) were purchased from Sigma (St. Louis, MO). Dulbecco’s modified Eagle’s medium (DMEM) was purchased from Gibco (Grand Island, NY, USA). Penicillin/streptomycin (P/S), phosphate-buffered saline (PBS), RIPA buffer, 0.25% trypsin-ethylenediaminetetraacetic acid solution and the Cell Counting Kit-8 (CCK-8) assay were provided by Solarbio (Beijing, China). PCR primers were synthesized by Biosune Biotechnology (Shanghai, China). The Evo M-MLV RT Kit with gDNA Clean for qPCR II, a SYBR Green Premix Pro Taq HS qPCR Kit and TRIzol reagent were purchased from Accurate Biotechnology (Beijing, China). An NO assay kit, a BCA protein assay kit, 4′,6′-diamidino-2-phenylindol (DAPI), a TdT-mediated dUTP nick-end labeling (TUNEL) kit, a reactive oxygen species (ROS) assay kit and a 5-ethynyl-2′-deoxyuridine (EdU) cell proliferation kit were purchased from Beyotime Biotechnology (Shanghai, China). The ECL detection kit was purchased from Meilunbio (Dalian, China).

The following primary antibodies were used: rabbit anti-VEGF-A (Abcam, Cambridge, MA, USA; ab252439), rabbit anti-eNOS (Abcam, Cambridge, MA, USA; ab52917), mouse anti-FXR (Santa Cruz, CA, USA; sc-25,309), rabbit anti-Bcl-2 (Cell Signaling Technology, Beverly, MA, USA; cat. no. 15,071), rabbit anti-Bax (Cell Signaling Technology, Beverly, MA, USA; cat. no. 2774) and rabbit anti-β-actin (BM0626, Boster, China). All the antibodies were used at a dilution of 1:1000 in Western blotting (WB) assays, and antibodies against VEGF-A (1:200) and eNOS (1:200) were used in immunofluorescence assays.

### 2.2. Cell culture

The MS-1 cell line (ATCC, Manassas, USA) was cultured in DMEM (Gibco, USA) supplemented with 5% FBS at 37°C in a 5% CO_2_ atmosphere. The cells were seeded at a density of 1 × 105 cells/well in 6-well plates for 24 h prior to further experiments.

### 2.3. Glucose exposure and metformin treatment

To expose the MS-1 cells to HG conditions, we cultured MS-1 cells with different concentrations of glucose, 5 mmol/L (mM), 12.5 mM, 25 mM, 33.3 mM and 50 Mm, for 24 h or 48 h. Then, we treated the cells that had been exposed to 33.3 mM glucose for 24 h with metformin at different concentrations (0 mM, 0.5 mM, 1 mM, 2 mM, and 5 mM) for different times (24 h, 48 h, 72 h, and 96 h). After the CCK-8 assay, we chose 2 mM as the best concentration of metformin for treatment and 48 h as the optimal intervention time.

### 2.4. Cell transfection

Small interfering RNA (siRNA) targeting FXR and VEGF-A was purchased from RiboBio (Guangzhou, China) and introduced into MS-1 cells with transfection kits according to the instructions [13]. Another group of cells was transfected with the control siRNA at the same concentration for the same transfection time.

### 2.5. Cell viability assay

The cytotoxic effect of glucose or metformin was evaluated using the CCK-8 assay [14]. MS-1 cells were seeded in a 96-well plate at a density of 2 × 10^3^ per well. Cell viability was assessed using the CCK-8 assay according to the manufacturer’s instructions. The absorbance at 450 nm was read with a microplate reader (Thermo Fisher, USA).

### 2.6. EdU staining

EdU staining was performed as previously described [15]. In short, treated MS-1 cells were incubated with an EdU working solution (10 μM) for 4 h at 37°C. Then, the MS-1 cells were fixed with 4% paraformaldehyde for 15 min. Subsequently, the cells were permeabilized with 0.1% Triton X-100 for 15 min and washed with PBS three times. Finally, the cells were incubated with DAPI for 5 min. Images were captured with a fluorescence microscope (Olympus, Japan). MS-1 cells undergoing DNA replication emitted red fluorescence, and the nuclei emitted blue fluorescence.

### 2.7. WB analysis

Total protein was extracted from MS-1 cells using a mixture of a protease inhibitor cocktail and RIPA buffer [14]. The protein concentration was quantified with a BCA kit. Proteins (20–40 µg) were separated by 10% SDS–PAGE and then electrotransferred onto polyvinylidene fluoride membranes. After blocking with 5% nonfat milk in TBST for 2 h, the membranes were incubated with primary antibodies overnight at 4°C. The membranes were incubated with secondary antibodies at a 1:10,000 dilution for 1 h. After the removal of the secondary antibody, the blots were washed and examined with an ECL detection kit. The proteins were imaged on an automated gel imaging analysis system and quantified using ImageJ software.

### 2.8. Real-time polymerase chain reaction (RT–PCR)

Total RNA was extracted from MS-1 cells using TRIzol, and cDNA was synthesized with a reverse transcription reagent kit according to the manufacturer’s instructions [14]. RT–PCR was performed with the SYBR Green PCR kit (Takara, Japan). Expression levels were calculated based on the 2− ΔΔCt comparative method and normalized to the expression of GAPDH. The primer sequences used are shown in Table 1.

**Table 1.** Primer sequences used in this study

Target	Sequences
FXR	Forward, 5’-TCGTTCGGCGGAGATTTTCA-3’
	Reverse, 5’-CTGTGAGCAGAGCGTACTCC-3’
Vegf-A	Forward, 5’-CCAAGATCCGCAGACGTGTA −3’
	Reverse, 5’-TTAACTCAAGCTGCCTCGCC-3’
Enos	Forward, 5’-AAGTGGGCAGCATCACCTAC-3’
	Reverse, 5’-CCAAGCAGCGTCTTGAGGTA-3’
GAPDH	Forward, 5’-TGTCTCCTGCGACTTCAACA-3’
	Reverse, 5’-GGTGGTCCAGGGTTTCTTACT-3’

### 2.9. Determination of NO levels

MS-1 cells were seeded in a 96-well plate at 4 × 10^3^ per well [16]. After treatment, 50 μL of cell supernatant or a standard was mixed with 50 μL of Griess Reagent I and II from an NO kit (Beyotime, Beijing, China). Then, the absorbance at 540 nm was measured with a microplate reader (Thermo Fisher, USA).

### 2.10. Immunofluorescence staining

MS-1 cells were grown on cover slips in a 24-well plate. After the treatment, the cells were fixed in 4% paraformaldehyde [17]. Then, the cells were blocked with 5% bovine serum albumin (BSA) diluted in 0.3% Triton X-100. MS-1 cells were incubated with a mixture of anti-FXR primary antibody and anti-VEGF-A primary antibody or a mixture of anti-FXR primary antibody and anti-eNOS primary antibody overnight at 4°C. After washing with PBS three times, the cells were incubated with a mixture of goat anti-rabbit IgG and goat anti-mouse IgG secondary antibodies in the dark at room temperature for 60 min. After staining with DAPI in the dark for 5 min, the cells were examined using a laser scanning confocal microscope (Leica, Germany).

### 2.11. Determination of ROS levels

Intracellular ROS production by MS-1 cells was assessed by measuring the fluorescence intensity of dihydroethidium staining [18]. After treatment, MS-1 cells were incubated with 10 μM dihydroethidium at 37°C for 30 min. Then, the cells were washed with PBS 3 times. Finally, the cells were immediately observed under a fluorescence microscope. ImageJ was used to quantify the fluorescence intensity of each image.

### 2.12. TUNEL staining

Apoptosis was determined by a TUNEL kit according to the manufacturer’s instructions [18]. MS-1 cells were cultured in 24-well plates. The cells were incubated with PBS supplemented with 0.5% Triton X-100 for 5 min. After adding TUNEL detection solution and incubating the cells at 37°C for 60 min, the samples were sealed with anti-fluorescence quenching solution and observed under a fluorescence microscope.

### 2.13. Statistical analyses

The data are expressed as the mean ± S.E.M. Statistical analyses were performed using two-way analysis of variance or Student’s t test (GraphPad Prism 8.0 software, San Diego, CA, USA). A P value<0.05 was used to indicate statistical significance.

## 3. Results

### 3.1. HG induced MS-1 cell apoptosis and oxidative stress and reduced cell viability

**Figure 1.**
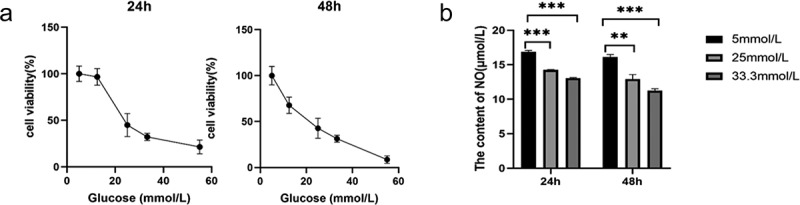
HG induced MS-1 cell apoptosis and oxidative stress and reduced viability. (a) The viability of MS-1 cells was observed at 24 and 48 h after treatment via CCK-8 assay. (b) Nitric oxide levels in the supernatants of MS-1 cells treated with HG were quantified. (** *p* < 0.01, *** *p* < 0.001).

To determine the optimal glucose concentration for reducing MS-1 cell viability, the cells were treated for 24 and 48 h at different concentrations of glucose. Cell apoptosis, oxidative stress, and viability as the indicators of cell damage are detected. Our results showed that after 24 h of treatment with 25 mM glucose, cell viability was obviously reduced, and 33.3 mM glucose considerably reduced cell viability compared with the control treatment. The negative effect of glucose on cell viability was more pronounced at 48 h. Then, we measured NO production in MS-1 cells treated with 25 mM and 33.3 mM glucose. NO production decreased as the glucose concentration increased when cells were treated for 24 h, and this effect was even stronger at 48 h (Figure 1(a)). ROS production was measured by dihydroethidium staining, and ROS production was distinctly increased in HG-treated MS-1 cells compared with control-treated cells (Supplementary Figure S1B). Furthermore, the present results from the WB analysis showed that Bax protein expression was evidently upregulated and Bcl-2 protein expression was markedly downregulated in the HG group (Supplementary Figure S1A). And, it was showed in TUNEL staining that HG treatment resulted in an increase in the percentage of TUNEL-positive MS-1 cells (Supplementary Figure S1C).

Based on the data shown in Figure 1 and Supplementary Figure S1, 33.3 mM glucose was found to be harmful to MS-1 cells, and glucose was used at this concentration for further experiments to determine the effects of metformin on damaged islet endothelial cells.

### 3.2. Effect of FXR on the endothelial dysfunction induced by HG conditions

**Figure 2.**
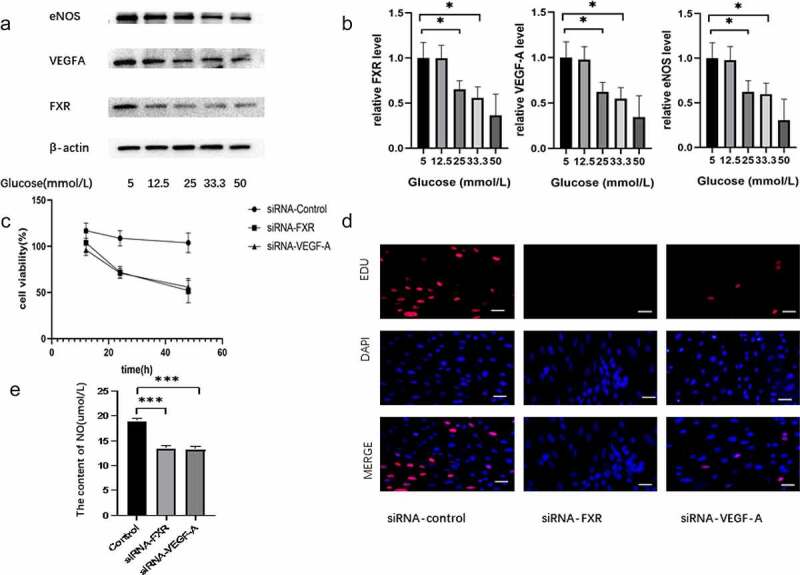
Effect of FXR on the endothelial dysfunction induced by HG conditions. (a) FXR, VEGF-A and eNOS expression was measured via WB analysis. (b) FXR, VEGF-A and eNOS mRNA expression was detected by real-time PCR. (c) The viability of MS-1 cells was observed after treatment via CCK-8 assay. (d) The proliferation of MS-1 cells was measured after treatment via EdU staining (Scale bar = 50 μm); (e) Nitric oxide levels were measured in the supernatants of MS-1 cells in which FXR or VEGF-A activity was inhibited (* p < 0.05, *** p < 0.001).

To investigate the effect of FXR on the endothelial dysfunction induced by HG, MS-1 cells were treated with medium supplemented with different concentrations of glucose for 48 h. And then the expression levels of FXR, VEGF-A and eNOS in MS-1 cells were detected by RT-PCR and WB. Subsequently, we used a genetic approach to inhibit FXR or VEGF-A activity and measured the proliferation, apoptosis and oxidative stress of MS-1 cells. Both WB and PCR results indicated that the expression of FXR and eNOS was markedly decreased in HG-treated MS-1 cells compared with normal control cells (Figure 2(a,b)). As illustrated in Figure 2(c,d), the viability and proliferation of MS-1 cells were markedly reduced in the presence of FXR siRNA or VEGF-A siRNA after 48 h of treatment. In addition, the decreasing of NO production was observed after inhibition of FXR or VEGF-A activity (Figure 2(e)).

### 3.3. Metformin ameliorated HG-induced MS-1 cell injury

**Figure 3.**
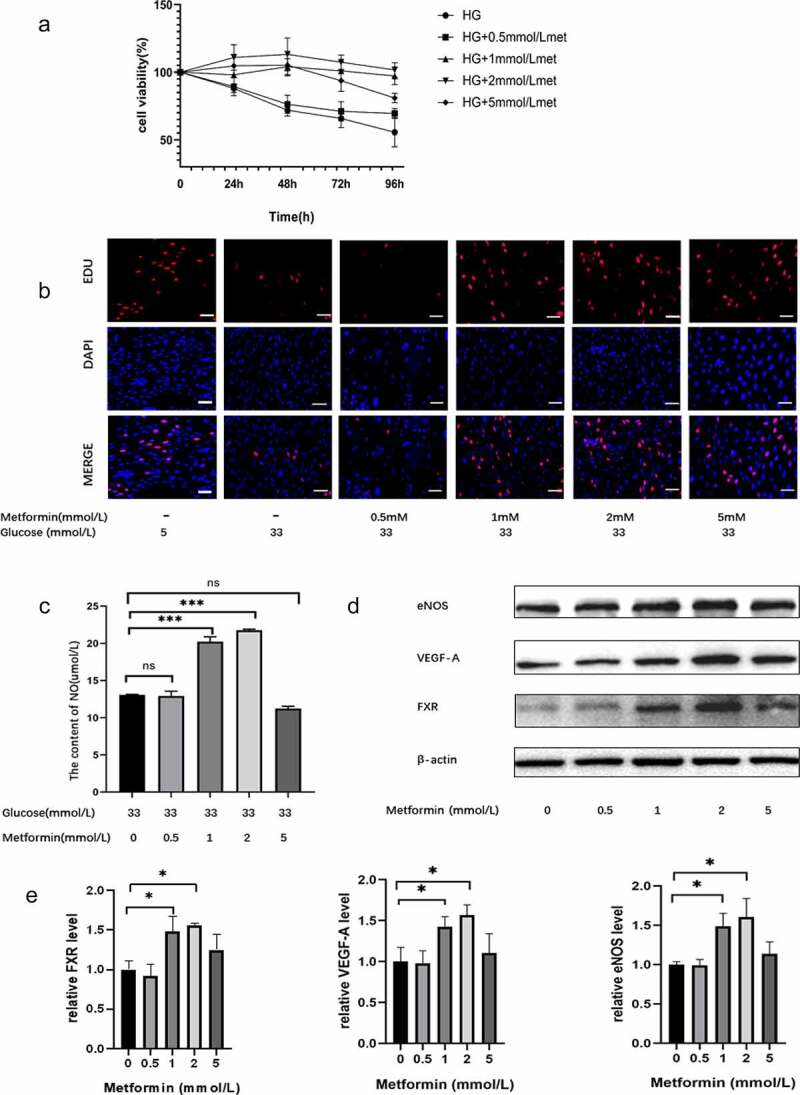
Metformin ameliorated HG-induced MS-1 cell injury. (a) The viability of metformin-treated MS-1 cells was observed after different time points via CCK-8 assay. (b) The proliferation of MS-1 cells was observed after metformin treatment via EdU staining (Scale bar = 50 μm). (c) NO levels were quantified in the supernatants of MS-1 cells treated with different concentrations of metformin. (d) FXR, VEGF-A and eNOS levels were detected via WB analysis. (e) FXR, VEGF-A and eNOS mRNA expression was detected by real-time PCR. (* p < 0.05, ** p < 0.01, *** p < 0.001).

To explore the effect of metformin on injured MS-1 cells, the cells were exposed to 33.3 mM glucose for 24 h and then treated with metformin for different times (24 h, 48 h, 72 h, or 96 h). Treatment with 33.3 mM glucose reduced cell viability, but this effect was reversed by metformin treatment. The results from the CCK-8 assay demonstrated that 2 mM is the optimal metformin concentration and that 48 h is the optimal time (Figure 3(a)). The effect of metformin on cell proliferation was also confirmed by EdU staining (Figure 3(b)). Additionally, metformin treatment reversed the changes in the NO and ROS levels in HG-treated cells (Figure 3(c) and supplementary Figure S2B). Similarly, metformin treatment significantly inhibited HG-induced MS-1 cell apoptosis, as demonstrated by TUNEL staining (Supplementary Figure S2C). And these effects were more pronounced after 48 h of treatment with 2 mM metformin. Moreover, metformin treatment elevated the levels of FXR, VEGF-A, Enos and Bcl-2 and reduced the level of Bax, thus reversing the effects of HG treatment, as expected. As shown in Supplementary Figure S2E and F, there was no significant difference in FXR, VEGF-A, Enos, Bcl-2 and Bax expression between the HG with 2 mM metformin-treated group and the NC group (5 mM glucose).

3.4. Metformin treatment ameliorated HG-induced injury by regulating the FXR/VEGF-A/eNOS signaling pathway

**Figure 4.**
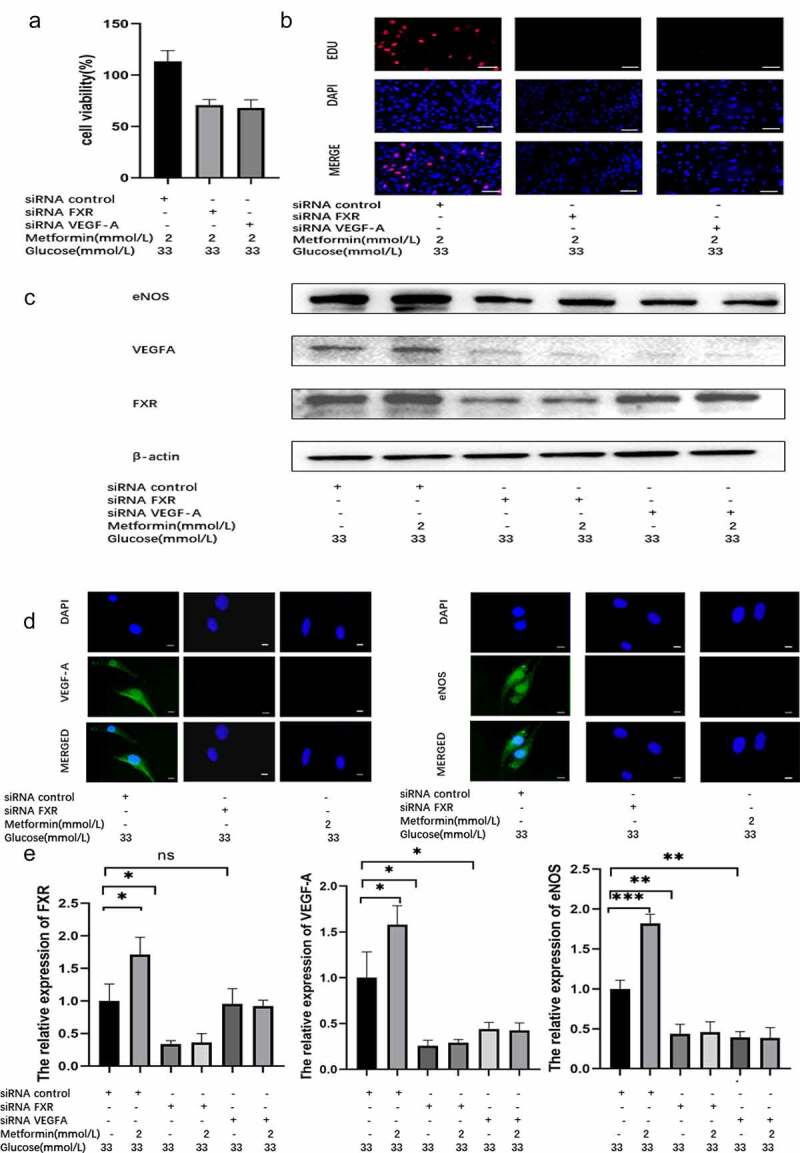
Metformin treatment alleviated HG-induced injury by regulating the FXR/VEGF-A/eNOS signaling pathway. (a) The viability of metformin-treated MS-1 cells in which FXR or VEGF-A activity was inhibited was observed after 48 h via CCK-8 assay. (b) The proliferation of metformin-treated MS-1 cells in which FXR or VEGF-A activity was inhibited was observed after 48 h via EdU staining (Scale bar = 50 μm). (c) FXR, VEGF-A and eNOS levels were detected via WB analysis. (d) FXR, VEGF-A and eNOS levels were detected via immunofluorescence (Scale bar = 20 μm). (e) FXR, VEGF-A and eNOS mRNA expression was detected by real-time PCR. (* p < 0.05, ** p < 0.01, *** p < 0.001).

To verify that the metformin-mediated amelioration of HG-induced injury depends on the FXR/VEGF-A/eNOS signaling pathway, we used a genetic approach to inhibit FXR or VEGF-A activity. CCK-8 assay, EdU staining and TUNEL staining results indicated that the protective role of metformin disappeared after FXR or VEGF-A activity was inhibited (Figure 4(a,b) and supplementary Figure S3C). Similarly, the reduction in ROS levels caused by metformin was reversed after FXR or VEGF-A expression was knocked down (Supplementary Figure S3B). WB analysis indicated that the FXR protein level was markedly suppressed in siRNA-FXR-transfected cells. Additionally, reduced protein expression of VEGF-A, eNOS, and Bcl-2 and increased protein expression of Bax were observed compared to those in the siRNA-control group (Figure 4(c) and supplementary Figure S3A). After metformin treatment, no significant difference in these protein expression levels was observed between the metformin + siRNA-FXR group and the siRNA-FXR groups. In addition, WB analysis demonstrated that the protein expression of VEGF-A and eNOS was obviously suppressed in the siRNA-VEGF-A-transfected cells. Although the protein expression of VEGF-A and eNOS was decreased, FXR protein expression was not significantly changed (Figure 4(c)). Then, we carried out PCR and immunofluorescence assays and obtained the same results (Figure 4(d,e)).

## 4. Discussion

To the best of our knowledge, this is the first study demonstrating the effects of metformin on islet endothelial cells. In this study, we showed that HG impaired the function of MS-1 cells, and metformin stimulated the proliferation and inhibited the apoptosis and oxidative stress of MS-1 cells. However, metformin potently reversed the harmful effects of HG. Moreover, HG downregulated the protein levels of FXR, VEGF-A, Bcl-2/Bax and eNOS in MS-1 cells, and metformin restored the levels of these proteins. In addition, genetic inhibition studies further showed that FXR can promote MS-1 cell proliferation and decrease the apoptosis and oxidative stress of MS-1 cells. The FXR/VEGF-A/eNOS signaling pathway is involved in the protective effects of metformin on HG-treated MS-1 cells.

The islets of Langerhans consist of approximately five different endocrine cell types and constitute the remaining 1% to 2% of the gland [19]. The islets are the only location where the blood glucose-lowering hormone insulin is produced; in addition, other islet-secreted hormones are involved in metabolism. Over the past several decades, diabetes has been shown to be characterized by impaired β cell function and insulin resistance [20]. Increasing evidence indicates that injury to IMECs is closely related to the occurrence and development of diabetes mellitus [21]. Therefore, it is necessary to elucidate the change in IMEC function under HG conditions and determine the effect of hypoglycemic drugs on these cells.

Several studies have demonstrated that HG conditions can cause endothelial dysfunction [22]. In the present study, 12.5–50 mM glucose was used to generate an injury model, and 33 mM glucose was used as the concentration used to treat the HG group. Previous studies have reported that these doses of HG could significantly increase intracellular ROS levels and induce IMEC apoptosis [23,24]. These studies found that 33 mM glucose can lead to an obvious increase in the levels of ROS. Additionally, increased oxidative stress is a critical factor in the development and progression of EC apoptosis.

Indeed, metformin has been shown to exert endothelial-protective effects independent of its hypoglycemic effect [25]. For example, metformin may prevent the endothelial dysfunction caused by hyperglycemia-related oxidative stress through Nr4a1 [26]. In our study, we found that metformin protects MS-1 cells through the FXR/VEGF-A/eNOS signaling pathway.

As a member of the nuclear receptor superfamily, both direct and indirect activation of FXR can ameliorate metabolic disease. Several studies have shown that FXR expression in islets can protect pancreatic islets [27]. In FXR-knockout mice, insulin secretion was decreased, although the β-cell mass was normal [28]. In addition, FXR has been suggested as a potential therapeutic target for treating endothelial dysfunction [29]. FXR can also activate AMP-activated protein kinase alpha (AMPKα), which plays distinct roles in inhibiting oxidative stress and reducing ROS production [30]. Furthermore, FXR can reduce the expression of Caspase-9 or Caspase-3 to inhibit apoptosis [31,32]. In the present study, we found that HG could decrease the expression of FXR in MS-1 cells. However, low concentrations of metformin, especially 2 mM, reversed this effect.

Inactivation of VEGF-A leads to severe loss of capillary density, vascular permeability and islet function in both endocrine progenitor cells and differentiated β cells [33]. Brissova, M. et al. demonstrated that regulation of the local islet microenvironment by VEGF-A signaling plays an indispensable role in β cell regeneration. This process relies on VEGF-A-mediated bone marrow-derived macrophage recruitment, which induces β cell proliferation either directly or in conjunction with the islet endothelium [34]. Additionally, VEGFA/VEGFA receptor (VEGFR) signaling is closely associated with cell proliferation and apoptosis [35]. Recently, Huang L. et al found that VEGF-A silencing can increase ROS levels in spinal cord cells and activate caspase-3 signaling to induce apoptosis [36]. In addition, inhibition of VEGF-A expression can amplify bevacizumab-induced oxidative damage, cardiomyocyte apoptosis, and ROS levels and can change the expression of related apoptotic proteins [37]. In our study, we showed that metformin can alleviate the HG-induced downregulation of VEGF-A expression in MS-1 cells.

Changes in NO metabolism are a common mechanism underlying endothelial dysfunction in cardiovascular diseases [38]. Previous studies have shown that bile acids can improve vascular endothelial function by increasing the release of NO, which regulates vascular permeability [39]. eNOS is a major source of NO in the vascular endothelium. The production of superoxide could interrupt the release of NO, resulting in considerable endothelial dysfunction [40]. In addition, eNOS uncoupling was found to lead to reactive oxygen species (ROS) production and induce oxidative stress in endothelial cells [41]. In the present study, HG decreased the protein and mRNA levels of eNOS in MS-1 cells, which was consistent with the finding that HG decreased NO generation. Notably, inhibition of FXR or VEGF-A function by siRNA abolished the protective effects of metformin on HG-treated MS-1 cells. These results suggest that metformin ameliorates the dysfunction of NO secretion and promotes the proliferation of MS-1 cells via the FXR/VEGF-A/eNOS signaling pathway.

This study has some limitations. First, we used only siRNA to test the involvement of the FXR/VEGF-A/eNOS signaling pathway in the protection of HG-treated endothelial cells. However, other methods, such as plasmid transfection into MS-1 cells, would be useful to confirm our discoveries. Second, we used only the MS-1 cell line as an in vitro injury model in this study. Animal experiments and/or primary mouse islet endothelial cell culture might provide more information to complement the current findings.

## 5. Conclusion

In summary, we found that metformin can prevent the MS-1 cell injury induced by HG conditions by alleviating oxidative stress, inhibiting apoptosis and promoting proliferation. Moreover, the protective effects of metformin may be mediated by activating FXR. In the future, we will further investigate the protective benefits of metformin and provide new evidence to further elucidate the underlying mechanism of metformin in the development of diabetic microcirculation disorders.

## Supplementary Material

Supplemental Material

## Data Availability

The raw data supporting the conclusions of this article will be made available by the authors, without undue reservation (http://dx.doi.org/10.1080/21655979.2022.2033411).
