# Supplementary material for: Metformin attenuates high glucose-induced injury in islet microvascular endothelial cells
Source: Bioengineered. 2022 Feb 9;13(2):4385–96. doi: 10.1080/21655979.2022.2033411 (PMC8973819; doi:10.1080/21655979.2022.2033411)
Supplement: Supplemental Material [file KBIE_A_2033411_SM9068.pptx]

## Slide 1
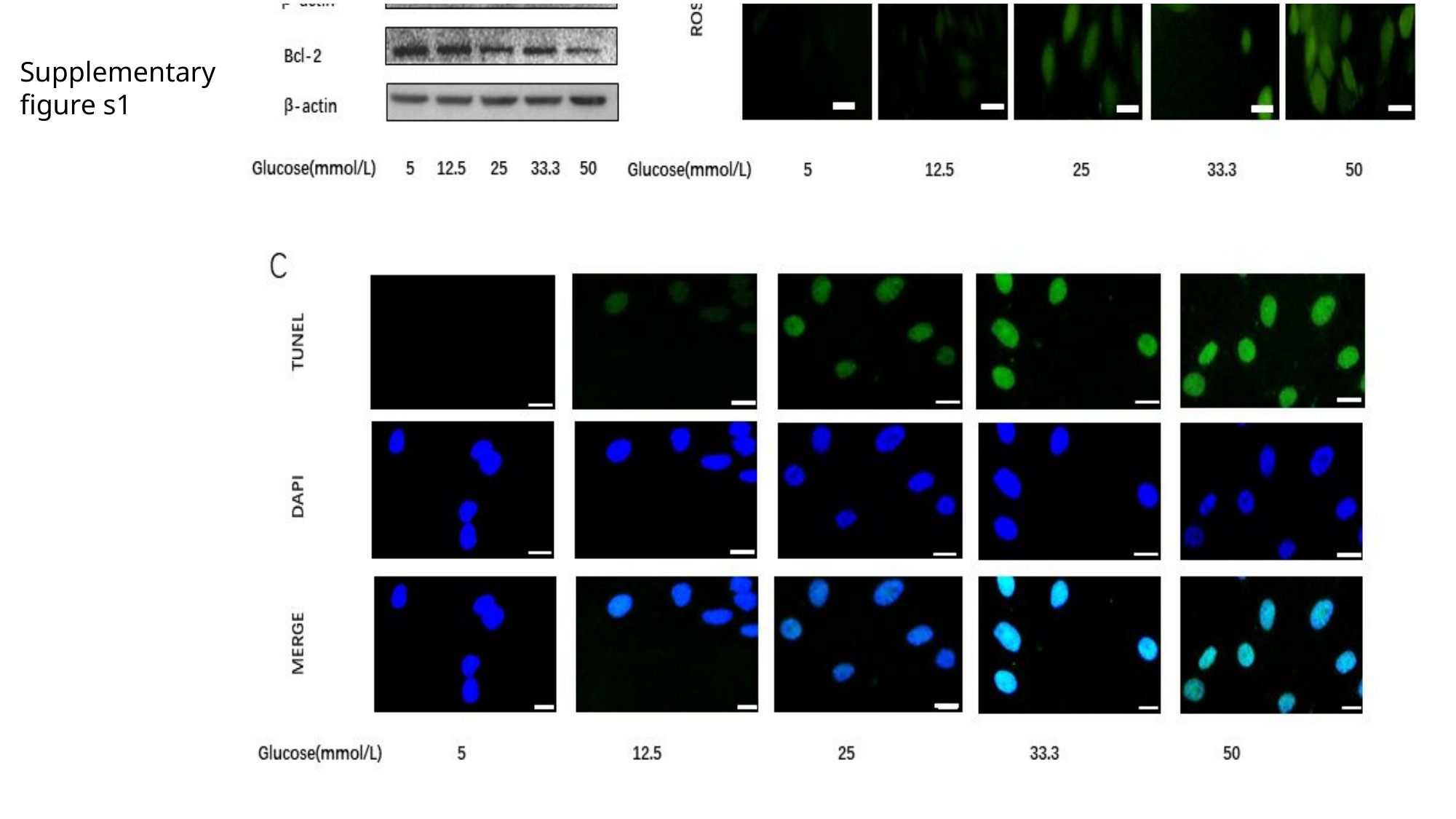

Supplementary figure s1

## Slide 2
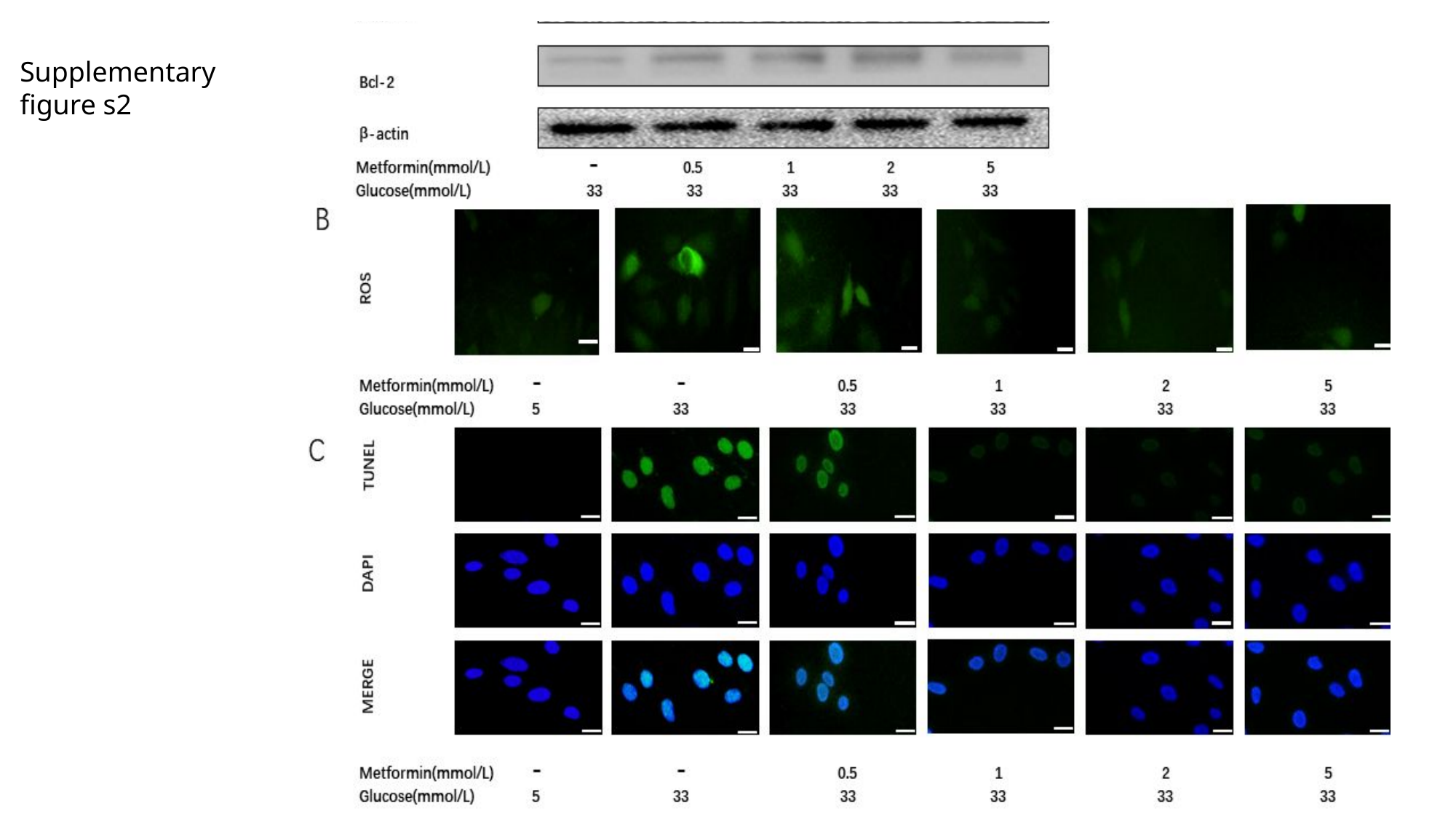

Supplementary figure s2

## Slide 3
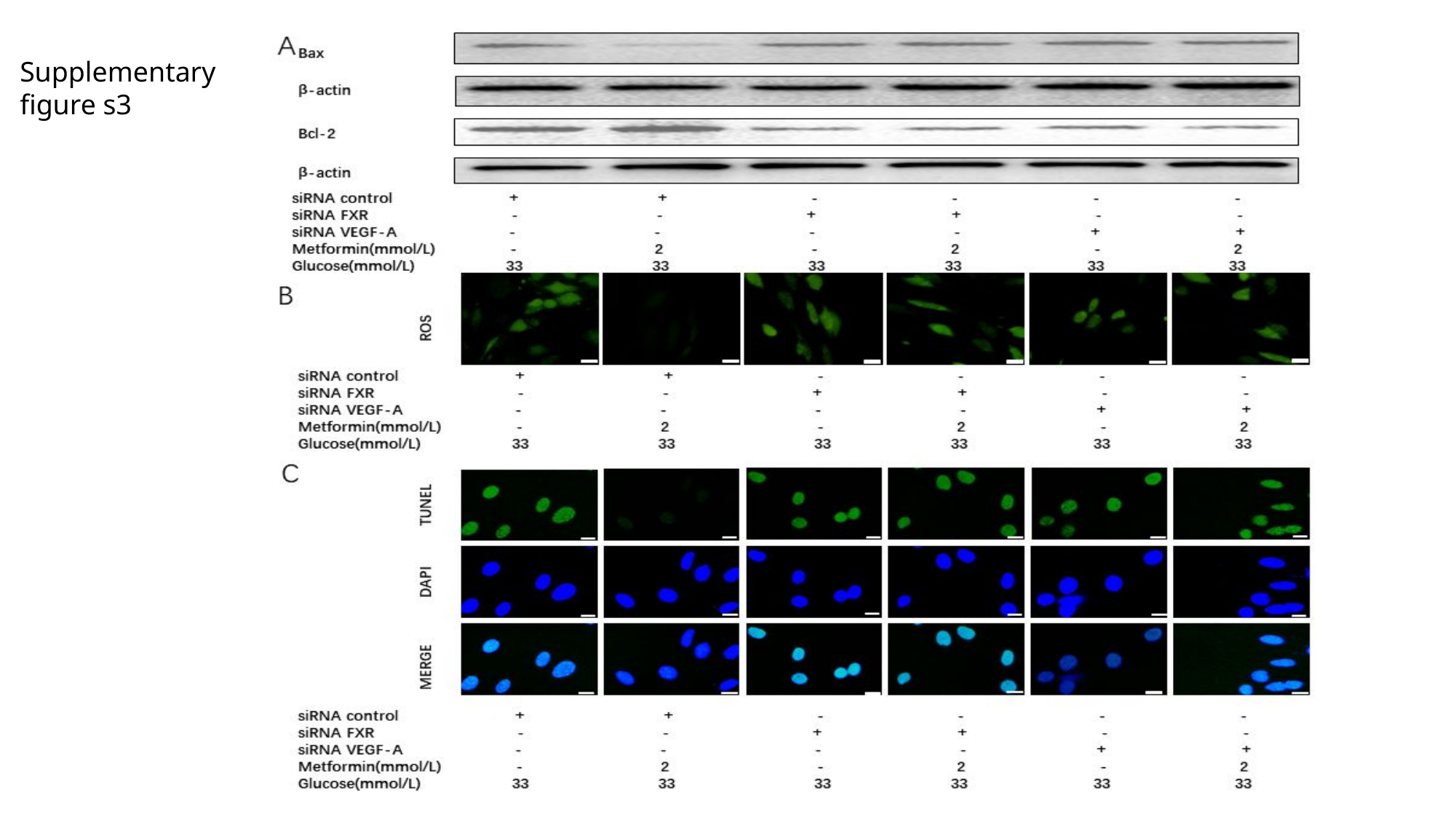

Supplementary figure s3

## Slide 4
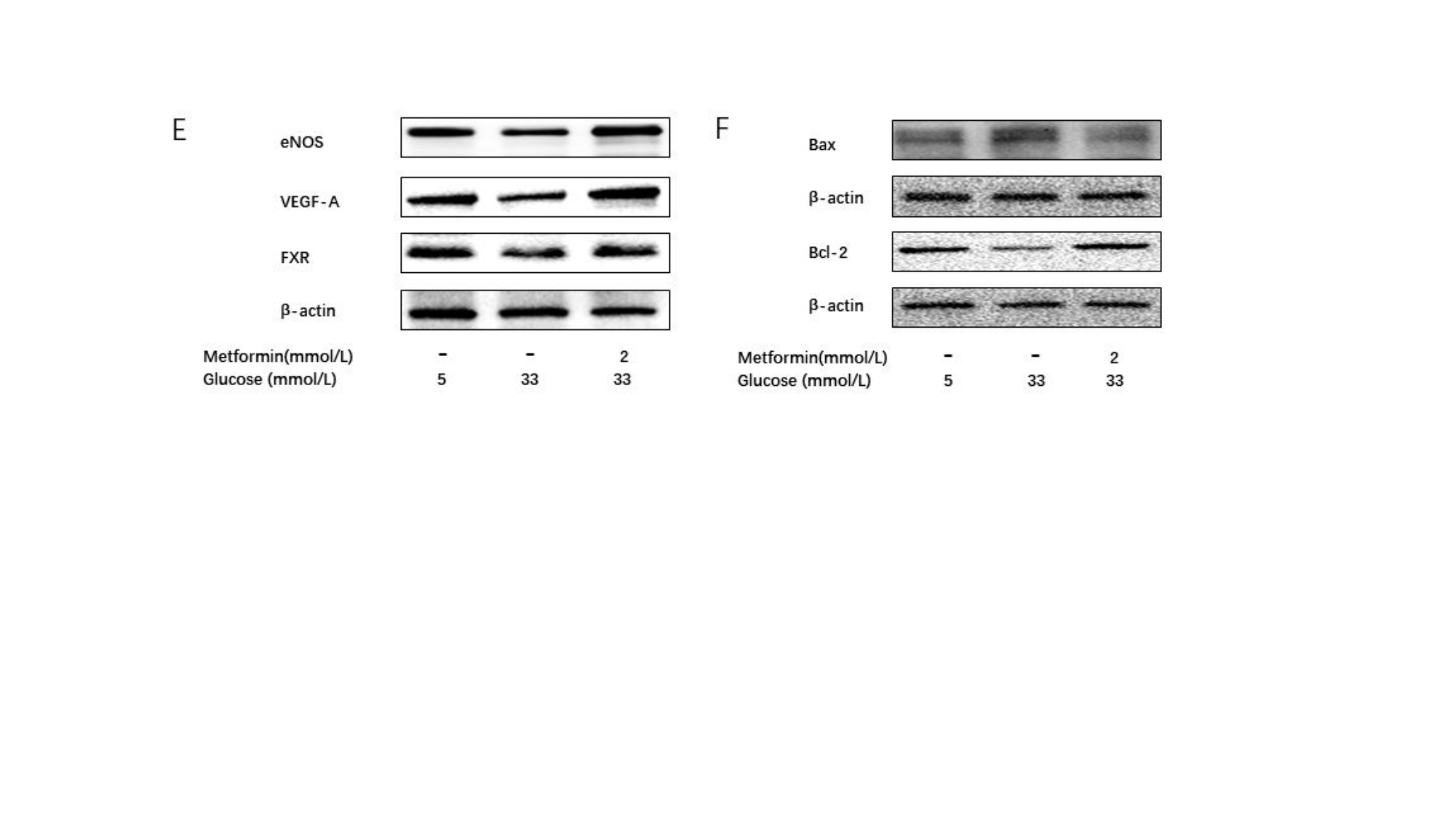

## Slide 5
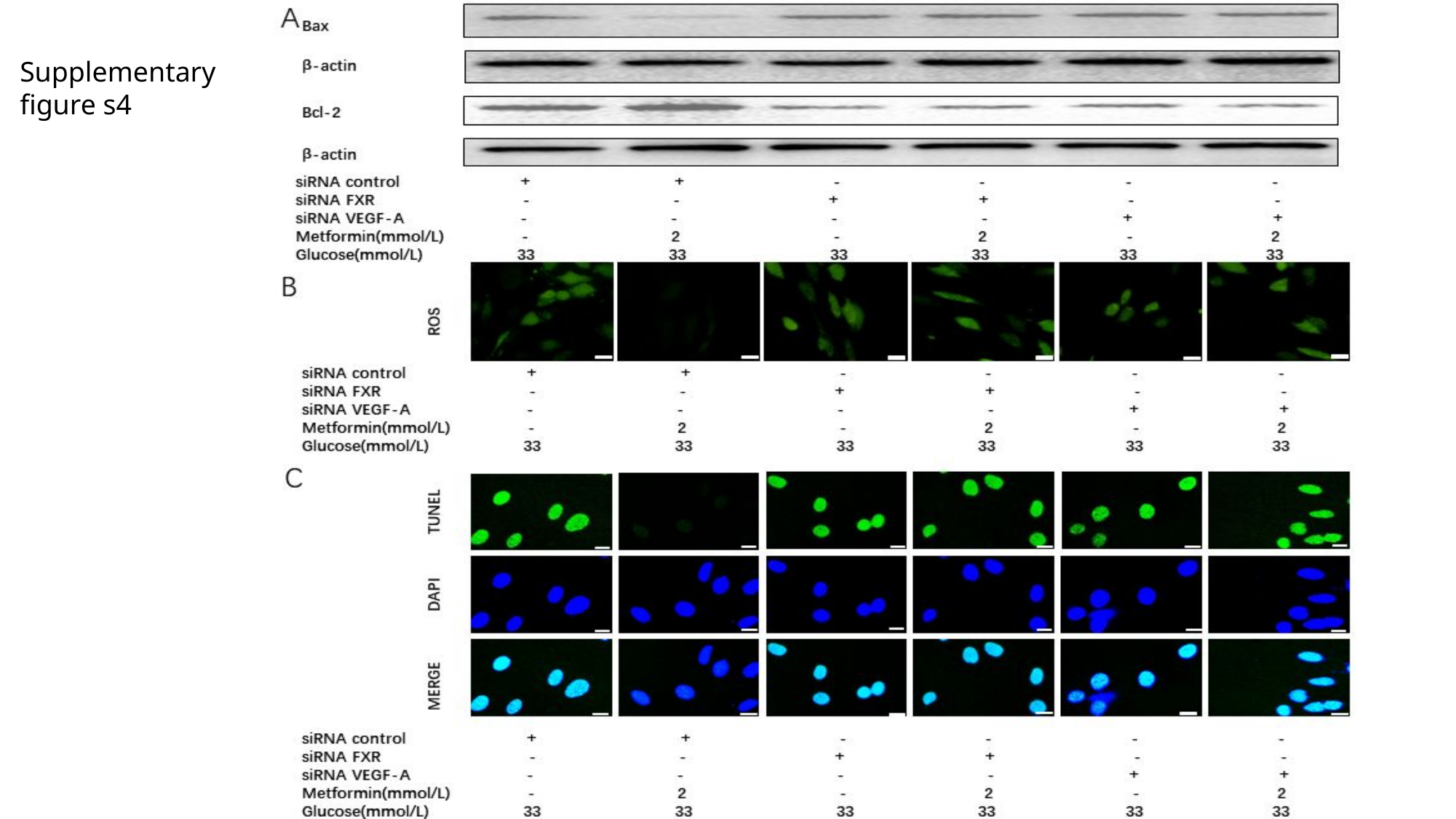

Supplementary figure s4
